# Current Perspectives on Gastrointestinal Models to Assess Probiotic-Pathogen Interactions

**DOI:** 10.3389/fmicb.2022.831455

**Published:** 2022-01-31

**Authors:** Mehreen Anjum, Arja Laitila, Arthur C. Ouwehand, Sofia D. Forssten

**Affiliations:** International Flavors and Fragrances, Health and Biosciences, Danisco Sweeteners Oy, Kantvik, Finland

**Keywords:** epithelium, gut, *in vitro*, microbiota, pathogen, probiotic

## Abstract

There are different models available that mimic the human intestinal epithelium and are thus available for studying probiotic and pathogen interactions in the gastrointestinal tract. Although, *in vivo* models make it possible to study the overall effects of a probiotic on a living subject, they cannot always be conducted and there is a general commitment to reduce the use of animal models. Hence, *in vitro* methods provide a more rapid tool for studying the interaction between probiotics and pathogens; as well as being ethically superior, faster, and less expensive. The *in vitro* models are represented by less complex traditional models, standard 2D models compromised of culture plates as well as Transwell inserts, and newer 3D models like organoids, enteroids, as well as organ-on-a-chip. The optimal model selected depends on the research question. Properly designed *in vitro* and/or *in vivo* studies are needed to examine the mechanism(s) of action of probiotics on pathogens to obtain physiologically relevant results.

## Introduction

The gastrointestinal tract is very complex with a quadruple layered structure; mucosa, submucosa, muscularis mucosa, and serosa. The mucosa can be defined as a layer of epithelial cells situated above the extracellular matrix (ECM)-rich lamina propria. On top of the epithelial cells may be a layer of mucus of varying thickness. The ECM provides physical structure for cells but also mechanical and chemical signals that are essential for different cellular processes (Hussey et al., 2017). The gastrointestinal tract has the highest concentration of microbes and contains an abundant and diverse microbiota distributed differently in the various parts of the system (Walter, 2008). It is a place for host-microbial, as well as bacterial-bacterial interactions and influences the outcome of health or disease (Dieterich et al., 2018). The bacterial interactions may include competition for nutrients and space, but also cross feeding, enabling the development of multispecies co-operation to facilitate mutual survival and growth in the gastrointestinal environment (Montalto et al., 2009). Further, the microbes play an important role in supporting host mucosal immunity and intestinal barrier function. Diet and other environmental factors have a big influence on the gut microbiota, and e.g., the use of fiber can help to maintain a healthy gut microbiota. However, this will not be discussed here further since the focus is on models for probiotic-pathogen interactions.

Different natural mechanisms exist in the host that prevent invasive bacteria from colonizing the host, such as gastric acidity, intestinal motility, destruction of bacteria by intestinal enzymes, bile, and release of immunoglobulin A. Any disturbance in these mechanisms would cause the commensal microbiota to change, with a potential increase in the pathogenic microbes causing dysbiosis (Ojetti et al., 2009; Littman and Pamer, 2011). For an infection to occur, the first step is the pathogen’s ability to adhere to the mucosal surface and compete with the residing intestinal microbiota (Collado et al., 2007). The commensal bacteria in the gut exclude other microorganisms by competing with them and adhering strongly to the receptor sites present in the intestinal tract, thus limiting the establishment of the incoming potentially pathogenic microbes and preventing infections (Preidis et al., 2011; Bermudez-Brito et al., 2012). Probiotics, defined as “live microorganisms that, when administered in adequate amounts, confer a health benefit on the host” (Hill et al., 2014), are good candidates for preventing gut infections by competing against invading pathogens. The genera *Bifidobacterium* and *Lactobacillus sensu lato* contain the most well characterized probiotic strains that are currently commercially available in the market (Fijan, 2014; Brodmann et al., 2017). Furthermore, new species are emerging (Rouanet et al., 2020).

A pathogen is any organism that can produce disease. Different types of pathogens exist, including members of viruses, bacteria, fungi, and parasites. Probiotic strains may compete with pathogens for adhesion to gastrointestinal receptors on epithelial cells and the overlying mucus layer. There are various mechanisms of actions that allow bacterial species to exclude one another such as bacteria-bacteria interactions at the attachment sites on the host-mucosal interface, and/or secretion of antimicrobial compounds, and/or competing for available nutrients (Bermudez-Brito et al., 2012; Plaza-Diaz et al., 2019).

**FIGURE 1:**
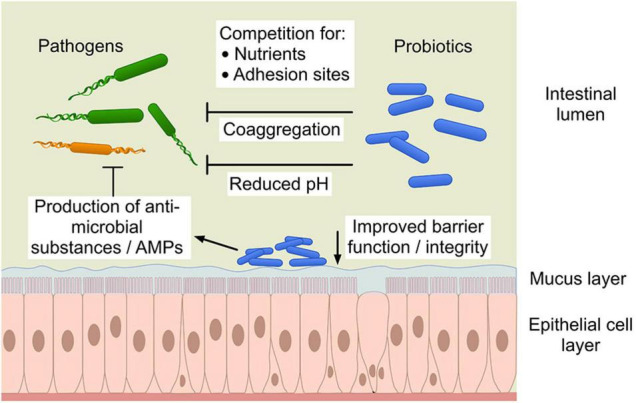
The different mechanisms of probiotic action against pathogens. “Copyright Pinja Kettunen/SciArt and IFF Health and Biosciences, with permission.” AMPs, antimicrobial peptides.

Probiotics may produce antimicrobial compounds (e.g., lactic or other short chain fatty acids, hydrogen peroxide or bacteriocins and low-molecular weight antimicrobial compounds) that can exert a direct effect on pathogens by inhibiting or killing them or by making the environment unsuitable for pathogen survival. Probiotics utilize nutrients and produce organic acids such as lactic acid, acetic, formic and succinic acid as primary metabolites that result in a lowering of the gut pH causing suppression of pathogen growth (Dicks and Botes, 2010; Bermudez-Brito et al., 2012). The different mechanisms of probiotics’ interaction with pathogens and host cells shows the complexity of elucidating these interactions (Figure 1). Moreover, these mechanisms are not only diverse but also mostly strain specific.

Since a probiotic should have demonstrable beneficial effects on the host, methods and techniques that would recreate physiologically relevant *in vitro* models that can simulate the *in vivo* environment, are important to study the interactions of commensal bacteria with epithelial cells as well as potential pathogens. The selection of a model must be done based on the hypothesis and mechanism of interest, and the culture conditions, cell types, as well as tools and methods need to be considered to select the most ideal biomimetic model. This review discusses the advantages and limitations posed by various models currently available to study host-microbe interactions within the gastrointestinal tract that can be translated to *in vivo* research.

## Approaches to Study Probiotic-Pathogen Interaction

### Animal Models

Animal models provide very controlled environments and enable the use of germ-free animals for investigating the interactions between host and microbe as well as potential pathogens. In addition, animal models provide the possibility to collect samples from different parts of the gastrointestinal tract that are not possible within clinical trials. The *in vivo* models applied in the probiotic research typically involve vertebrate laboratory animals, most commonly mice and rats. Mice are one of the most frequently used models because their intestinal development is similar to the human intestine and they also have many of the same immune responses and genes (Chinwalla et al., 2002) but some of their intestinal responses to inflammation may differ from human response (Seok et al., 2013). Even though the anatomy of gastrointestinal tract in mice and humans is similar, there are distinctions, e.g., the proportionally larger colon and cecum surface area and taller intestinal villi in mice (Park and Im, 2020). In addition, although the human and mice gut microbiota have 90 and 89% similarities in phyla and genera (Krych et al., 2013), there are differences in the abundance of microbes; especially lactobacilli and bifidobacteria (Park and Im, 2020). Another widely applied model is rats. Rats being larger than mice provide the advantage of providing larger samples. Mice are easy to breed with short gestation period and have large litter sizes, all of which contributes to their ease-of-use (Nguyen and Xu, 2008). These animal models being close to humans genetically and physiologically have some advantages for investigating probiotic-pathogen interaction. However, it is important to consider the similarities and differences between their intestinal microbiota when drawing conclusions. Mice and rats are coprophagic animals which can have an impact on the diet-based intervention (Jiminez et al., 2015).

**FIGURE 2:**
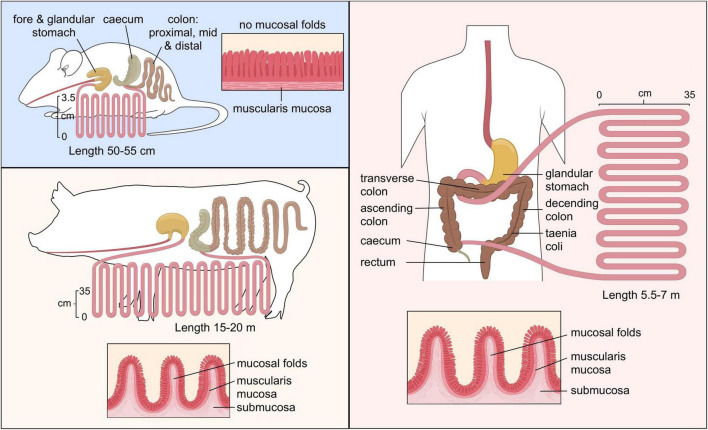
Comparison of the gastrointestinal tract of murine, pig and human. “Copyright Pinja Kettunen/SciArt and IFF Health and Biosciences, with permission.”

Another animal model is the pig. While the murine gastrointestinal tract is different from human, the porcine and human intestinal physiology and function are very similar with respect to anatomical and physiological characteristics (Figure 2), including digesta transit times, digestive and absorptive processes (Heinritz et al., 2013; Sciascia et al., 2016). While mice and humans have Paneth cells predominantly in the small intestine, the existence of the Paneth cell in the pig remains disputed (Gonzalez et al., 2013). The Paneth cells contain antimicrobial peptides and immunomodulating proteins that regulate the composition of the intestinal microbiota. However, the digestive enzyme and intestinal microbiota of a pig are comparable to humans which makes them good candidates for studies investigating microbial relationships and diet-based interventions (Patterson et al., 2008). Furthermore, there are big drawbacks as well, such as financial cost of conducting research in these models, the possibility of infection from a potential zoonotic microbes and ethical concerns (Coors et al., 2010).

### Alternatives to Animal Models

There are several ethical concerns connected to the use of animals in research, and the 3 R guiding principles were already described in 1959 for using animals in research and have evolved since then (Tannenbaum and Bennett, 2015). The 3 Rs: replacement, reduction and refinement revolve around the idea of ideally eliminating the use of animal models or decreasing as much as possible as well as treating animals humanely when these models are absolutely needed. *In vivo* testing of a probiotic strain maybe necessary for scientific and regulatory purposes before the strain can be accepted for widespread use in humans or animals. However, initial screening and selection of probiotic strains, their mechanism of action and health benefits can be studied using the *in vitro* models.

*In vitro* models have been developed for the pre-selection of potential probiotic strains due to the difficulties associated with *in vivo* studies in humans. By using *in vitro* systems, the physiological and functional properties of microorganisms against pathogens as well as various conditions in the digestive tract such as low pH, pepsin, pancreatic enzymes, bile salts, lysozyme as well as their ability to bind to epithelial cells can be tested (Kos et al., 2003). Table 1 summarizes the advantages and disadvantages associated with the broad categories of *in vitro* and *in vivo* models discussed below.

**TABLE 1:** Comparison of the advantage and the limitations of models for studying bacteria-pathogen and bacteria-host interaction.

Type of model	Examples of models*	Advantages	Limitations	Example references
“Simpler” *in vitro* methods	Agar spot test, broth microdilution, *in vitro* biofilms	Fast, inexpensive, high throughput, easy-to-perform Flexibility to change parameters easily Wealth of literature available for comparison Standardized protocols across laboratories Good for initial screening purposes	Does not represent *in vivo* responses Host response is missing Oversimplified models	Balouiri et al., 2016; Fijan, 2016
2D models	Caco-2, HT-29, HT29-MTX, T84, IEC-18 and IPEC-J2 tissue culture cells	Reproducible in lab environment Easy-to-perform Good for initial screening Simple model; well characterized in literature Several visualization methods have been optimized for 2D models	Cell lines mostly derived from cancer cells, thus different from healthy cells Does not include most cell types Hard to culture obligate anaerobes in co-culture due to oxygen requirements Grown as monolayer so they lack 3D structure	McGuckin et al., 2011; Vergauwen, 2015; Devriese et al., 2017; Jose et al., 2017; Yang et al., 2017; Gharbi et al., 2019
3D models	Organoids (e.g., enteroids and colonoids)	Mimics *in vivo* conditions Multicellular model Possibility of long-term cultures Possibility to investigate cell-cell interaction	Expensive and requires specialized expertise May, need biopsy/tissue samples Variability between models Difficult to study obligate anaerobes because of oxygen requirement Absence of shear forces and intestinal peristaltic movements to help cell differentiation Require complex media formulation and supplements	Werner et al., 2016; Costa and Ahluwalia, 2019; Bédard et al., 2020; Zhang et al., 2020
Chip based models	Microfluidic and multi-channel models	Non-transformed cell lines used and includes all cell types Patient specific tissue biopsies can be used to simulate disease conditions Peristalsis like movement can be included to enhance cell differentiation	Requires access to tissue biopsies Expensive, laborious and requires specialized expertise Variability between donors Small sampling size for downstream analysis	Ingber, 2016; Bein et al., 2018; Sontheimer-Phelps et al., 2020; Baddal and Marrazzo, 2021
*In vitro* digestive models	Fecal batch-culture, SHIME, TIM, Enteromix, Reading, PolyFermS	Allow the study of interactions with intestinal microbiota Study microbiota from different populations Include other models to study host interaction	No or limited ethical concerns Usually fecal inoculum Expensive to run	Minekus, 2015; Van de Wiele et al., 2015; Lamichhane et al., 2016; Piatek et al., 2020
*In silico* models		Fast forecast of host-microbiota-probiotic interactions	Only as good as theoretical knowledge of interactions	Geng et al., 2021
*Ex vivo* models	InteTESTine, Ussing chamber, IVOC	*In vivo* like multilayered structure Can be used to investigate bacteria interaction in diseased organs” Results can be translated to *in vivo* conditions	Requires access to human tissue biopsies and can be difficult to obtain Costly Tissues obtained need to be viable and fresh	Haque et al., 2004; Randall et al., 2011; Tsilingiri et al., 2013; Van Krimpen et al., 2014; Newburg et al., 2016; Thomson et al., 2019
Animal models	Mice, rats and pigs	Physiological model Allows investigation in the presence of intact gut microbiota with host cell interactions Possibility to conduct long term investigations Microbiota can be manipulated through diet Innate and adaptive immune response similar to humans	Ethically concerning Requires housing and care In some cases, cannot be translated to human responses Expensive experiments	Heinritz et al., 2013; Jiminez et al., 2015; Sciascia et al., 2016
“Simpler” animal models	*C. elegans*, honey bee, *Ciona robusta*, fruit fly, greater wax moth	Relatively fast, inexpensive Includes host response May include defined microbiota	Low/no ethical concerns Simplified host physiology Simplified microbiota	Vilela et al., 2015; Zanni et al., 2015; Zheng et al., 2018; Kumar et al., 2020; Poupet et al., 2020; Liberti et al., 2021

*Please see text for more detailed description of the models.

### Traditional Methods for Studying Probiotic-Pathogen Interaction

The antimicrobial activity of probiotics has been mainly studied using the traditional microbiological cultivation assays methods (Silva et al., 2020). The antagonistic activity of probiotics against a pathogen has been determined either through direct bacteria-bacteria interaction or by using cell free supernatant of probiotic bacteria (Fijan, 2016; Piatek et al., 2020). The assay to determine the antimicrobial activity can be performed on solid media (Balouiri et al., 2016) such as the agar spot test (Fijan, 2016). The spot assay may be employed to determine the direct effect by growing the probiotic and pathogen together and the pathogen will be inhibited by the release of inhibiting substance at the start of culture growth. A disadvantage of these cultivation-based methods is that the growth of the probiotic may lead to acidification of the environment and any inhibition observed is attributable to this. Another way is to determine the antimicrobial effect by allowing the probiotic spot to grow on agar media before it is inactivated which is then overlaid with the pathogen mixed in molten agar on top of the probiotic. The antimicrobial activity is determined by calculating the diameter of inhibition area (halo) around the probiotic spot.

A further alternative is the agar well diffusion assay to determine the antimicrobial activity, using cell free supernatants. In this case the pathogen is grown on the agar plate. Small holes of about 6 mm are punched in the agar which are filled with different concentrations of the cell free supernatant of the probiotic. The antimicrobial activity is measured using the inhibition zone around the well (Parente et al., 1995). The advantage of this method is that the supernatant can be neutralized to avoid the acid effect against the pathogen to study other antibacterial property. Microdilution method is a standardized method for testing antimicrobial activity. Different dilutions of probiotic cell free supernatant of are used in a liquid growth media. A standard inoculum of pathogen is added to it and incubated under appropriate conditions. The broth microdilution method is used to obtain minimal inhibitory concentrations values of the antimicrobial reagent (Balouiri et al., 2016). Convenient as they are, these methods are simplistic in their set up and do not account for the fact that an important ability of the microorganisms is to develop as biofilms (Flemming et al., 2016).

Several *in vitro* studies have documented the antimicrobial effect of probiotics against various human pathogens. A range of multi-strain probiotic products (*Saccharomyces cerevisiae* var. *boulardii, Lacticaseibacillus rhamnosus* GG or *Limosilactobacillus reuteri*) were screened using traditional plate cultures for studying their antagonistic properties against pathogens such as enteropathogenic *Escherichia coli* (EPEC), *Shigella, Salmonella, Klebsiella pneumoniae*, and *Clostridioides difficile* to support the selection process of products for further clinical evaluation (Piatek et al., 2020). In addition, e.g., the inhibitory effect of probiotic species of *Lactobacillus sensu lato* against *Staphylococcus aureus*, *Enterococcus faecalis, K. pneumonia, Pseudomonas aerugenosa*, and *Salmonella typhii* have been evaluated (Prabhurajeshwar and Chandrakanth, 2019), as well as *Bifidobacterium bifidum*, *Bacillus*, *L. reuteri, L. rhamnosus* GG, *Propionibacterium freudenreichii, Propionibacterium acnes, Lacticaseibacillus paracasei, Lactiplantibacillus plantarum, Lactobacillus bulgaricus*, were shown to inhibit pathogens such as *S. aureus* and *K. pneumoniae* (Orsi et al., 2014; Lagrafeuille et al., 2018).

A more recent batch fermentation method simulating the distal colon could potentially be used for studying probiotic pathogen interactions, however so far only *E. coli* Nissle have been tested in this model with simplified communities of gut bacteria (Arcidiacono et al., 2021).

### Human Gut Associated Biofilm Models

It is well established that in most biological systems microbes exist as multispecies biofilm layers instead of single species in free living state. Biofilms are present in the gastrointestinal tract where polymicrobial biofilms naturally grow at the mucosal surface as well as in the lumen as mucin-attached and food particle-attached colonies (Motta et al., 2021). The biofilm formation of probiotic bacteria can be beneficial as it may allow them to survive longer in the intestine and counteract colonization by enteropathogens. Lactic acid bacteria producing bacteriocin have been shown to be good candidates to develop protective biofilms to compete and displace pathogenic bacteria (Gomez et al., 2016; Perez-Ibarreche et al., 2016). Thus, to study and explore mechanisms of bacterial interactions, the experimental systems used *in vitro* ideally must be able to replicate the biofilm environment (Coenye and Nelis, 2010; Lebeaux et al., 2013). However, there are very few studies focused on screening antibiofilm activity of probiotics and even though some researchers have studied this biological activity of probiotics, their methods and analysis have varied (Cui et al., 2018; Hager et al., 2019; Silva et al., 2020).

Some of the advantages of using *in vitro* biofilm models are low cost, reproducibility, high throughput investigation and flexibility to obtain conditions that allow growth of the microorganisms being tested. One of the disadvantages of *in vitro* models is that they represent an oversimplification of the *in vivo* environment because it is hard to include, e.g., the immune response of the host *in vitro*. The challenge in co-culturing is to understand how the microbes would behave together as one species may end up outcompeting or even killing the other species, even though they coexist in a natural environment stably. Finding the right growth media and environment that would support simultaneous growth of all species under investigation is challenging. To make things more complicated, it is even more challenging to adjust growth rates *in vitro* to ensure that one species is not outgrowing the other artificially just because the chosen conditions support its growth more. A further challenge with *in vitro* multi-species biofilm models, is visualization of one species or its isolation from the complex environment (Coenye and Nelis, 2010; Lebeaux et al., 2013).

The existing biofilm models can be broadly categorized into static and dynamic models (Motta et al., 2021). Static models are closed systems, like microtiter plates, and these are low in cost, flexible and provide high throughput systems. While easy to use, these models are more limited in translation to *in vivo* environments. In static models the growth media and surface material can be modified to optimize the biofilm formation but since they are closed systems the nutrient supply is limited and metabolites accumulate; thus, they do not allow experiments to be run for long durations. In addition, the surface material used for bacterial adhesion in static models is synthetic, mostly plastic which is not ideal to study infection mechanisms. However, more biologically relevant materials, such as extra cellular matrix proteins, could be used as substratum. Many of the biofilm models have been used for oral and wound biofilm formation, but also for some probiotic-pathogen interactions. For example, the formation of *Vibrio*-biofilm was studied by microtiter plates to which cell free culture supernatant of lactobacilli were applied and hence the biofilm formation of *V. cholerae* was inhibited (Kaur et al., 2018). Some examples of static models that have been used for probiotic-pathogen interactions are multi-well microtiter plates (Gabrilska and Rumbaugh, 2015), the Calgary biofilm device for determination of antibiotic susceptibilities (Ceri et al., 1999) and Agar based static models (Guantario et al., 2018).

In contrast to static biofilm models, dynamic or open-system models provide a continuous supply of nutrients to the microbes in the biofilm (Gabrilska and Rumbaugh, 2015). These models are less cost-efficient than static systems and are not meant for high throughput experiments. Rather, the dynamic models are designed to mimic the actual host environment; thus, nutrients can be provided continuously, and shear forces present in natural conditions, can be modeled. The constant supply of nutrients allows the biofilm formation to survive for longer periods. The shear forces and the material that generates them can define the development of the biofilm by affecting the physical and chemical properties. However, these models have not been used to study probiotic-pathogen interaction in the gastrointestinal tract, more for, e.g., oral cavity (Salli et al., 2017) and urinary tract (Azevedo et al., 2017) environments.

### Two-Dimensional Intestinal Cell Models

Nearly all two-dimensional (2D) cultures are dependent on adhesion and thus cannot be grown in suspension cultures without mechanical support. Different cell lines such as primary or transformed cells can be used for 2D cultures, and cell lines like Caco-2 (Jose et al., 2017), HT-29 (Gharbi et al., 2019) and T84 (Devriese et al., 2017) have been used to understand bacterial survival and replication as the cells provide the host-like intestinal microenvironment. The small and large intestine have critical roles in the absorption of nutrients as well as house a large part of the human microbiome. During the past decade, 2D model systems comprised of culture plates as well as Transwell inserts have attempted to recapitulate the complex, *in vivo* intestinal physiology using cell lines derived from intestinal tumors instead of primary epithelial cells. In order to decide which cell lines to use, certain factors should be assessed, such as the culture condition required, media preparation, differentiation of cells needed, number of passages required and the expression of genes and proteins needed for the investigational purpose. There are advantages and disadvantages of using some of the most used cell lines in research and that will be discussed forthwith.

The classical static culture system mainly generating adherent 2D cell monolayer has several advantages; they are easy to maintain and the results derived are reproducible and consistent (Duval et al., 2017). Transformed or immortalized cell lines have been used extensively as they are cost-effective and enduring models. These cell lines can be passaged indefinitely and have been utilized extensively to perform preliminary screening and mechanistic studies. In addition, high throughput screening and imaging techniques are available, although *in vivo* characteristics may not be represented accurately (Joshi et al., 2019). It is the simplicity and efficiency of 2D cell lines that have made them so popular for *in vitro* studies (Bédard et al., 2020). On the other hand, primary cell lines are often considered to be more biologically and physiologically similar to the *in vivo* situation (Shamir and Ewald, 2014).

The disadvantage of using immortalized cell lines is that they cannot mimic the actual infection process completely as they lack the complexity of all cell types present *in vivo* as well as the immune responses (Finlay and Brumell, 2000). Since the cell responses are different, it also affects biochemical and biomechanical properties of cells (Edmondson et al., 2014). The 2D cultures of immortalized cells only have one cell type, making it difficult to mimic the complex architecture of the *in vivo* mucosa. This lack of cellular complexity compared to *in vivo* conditions is a serious disadvantage of 2D models available.

Even though the 2D cell models have been in use for decades, yet there is not a standardized *in vitro* model that has incorporated non-cancerous cell lines to study healthy large intestine response to bacterial interaction, which makes translation of response to healthy individual difficult. Thus, so far, the models applied do not have the ability to incorporate a complex bacteria community and have hence been usually used for investigating single bacterium-host interaction. Cells are grown on flat surfaces which causes them to behave differently as it leads to an unnatural polarity on the apical and basal surfaces of the cells which is especially an issue for cells that are non-polar *in vivo* (Bédard et al., 2020).

One challenge with cell models, is co-culturing of probiotic or intestinal bacteria and intestinal epithelial cells since the cells require aerobic conditions, while many of the bacterial strains require anaerobic conditions (Kim et al., 2010; Shin et al., 2019), although some recent efforts have been made to improve co-culturing (Shin et al., 2019; Sasaki et al., 2020).

Caco-2 cells grown as confluent monolayers have been used to investigate absorptive and transport kinetics under basal and bacteria exposed conditions. The disadvantage of using Caco-2 cells is that they originate from cancer cells and they lose their original characteristics during long culturing processes forming derivative cells (Foulke-Abel et al., 2014; Hoarau-Véchot et al., 2018). They are also unable to produce similar level of mucin under lab conditions as *in vivo* (Pan et al., 2015). However, one option is to incorporate a mucin layer to the Caco-2 cells monolayer which in some studies gave a better approach to the *in vivo* physiological characteristics (Santbergen et al., 2020).

More than 200 research papers have applied Caco-2 models to study intestinal barrier function and bacterial adhesion and invasion properties (Pearce et al., 2018). The protective role of different lactobacillus species, e.g., *L. rhamnosus* GG and *L. casei* against inflammation (Toki et al., 2009), and *L. plantarum* against *C. sporogenes* and *E. faecalis* adhesion (Ramiah et al., 2008) have been evaluated by using Caco-2 models. The challenge here is that tissue culture cells require aerobic conditions while many bacteria require anaerobic conditions; further, simulation of the intestinal environment would require anaerobic or microaerobic conditions. Chip based models may be better suited to simulate this; see below (Jalili-Firoozinezhad et al., 2019; Poceviciute and Ismagilov, 2019).

Another popular cell line that contains a mucus layer is HT-29. HT-29 cells have similar drawbacks as Caco-2, as they also are transformed cells derived from colon cancer cells. In addition, HT-29 cells are unable to form proper tight junctions thus, cannot be used to study barrier function (Schoultz and Keita, 2020), However HT29cl.f8, derived from a single cell of HT29 display important characteristics for permeability studies such as microvilli, tight junctions and a high transepithelial/transendothelial electrical resistance (TEER) (Mitchell and Ball, 2004). Hence, this clone can be an alternative to model the intestinal barrier (Sun et al., 2019).

By treating HT-29 cultures with methotrexate, mucus-secreting cells (HT29-MTX) have been obtained (McGuckin et al., 2011), and these cells exhibit entirely differentiated goblet cell-like phenotype and secrete low amounts of mucin (MUC2) that is predominantly expressed in the small and large intestine. The HT29-MTX cell model has more physiologically relevant characteristics because of the mucus layer formation than the HT-29, and may thus be better suited for studying cells-pathogen/probiotic interactions (Gagnon et al., 2013). Bacterial adhesion to the epithelium can be studied by using HT-29 cells that contain mucus-producing goblet cells in Transwell system with apical and basolateral polarity (Altamimi et al., 2016). HT-29 cells have also been used to study the inhibitory effect of *Lactobacillus acidophilus* NCFM, *L. rhamnosus* and *L. casei* on intestinal pathogens like *Salmonella* and *E. coli* (Toki et al., 2009; Meng et al., 2017).

To obtain more physiologically and functionally relevant results for probiotic-pathogen interactions, co-cultures of Caco-2 and HT-29 cells can be used. However, this model has more commonly been used for studying adhesion properties of probiotic strains rather than probiotic-pathogen interactions. One study using co-culture exposed the cells to *Streptococcus thermophilus* and *L. acidophilus* and as a result the barrier function of the cell monolayer was enhanced and provided protection against invasion and adhesion of enteroinvasive *E. coli* (EIEC) (Resta-Lenert and Barrett, 2003).

A non-transformed cell line IEC-6 that originates from epithelial cells of small intestine from *Rattus norvegicus* (rat) are an example of healthy cell type. A drawback of these cell lines though is that they do not depict a similar metabolic or absorptive response as human cells including the colon physiology. Hence, the interaction of bacteria to epithelium is different which makes translation of research conducted in these cell lines to human response difficult. The IEC-6 have more been used to study the effect of probiotics on different conditions like necrotizing enterocolitis (NEC), stress (Khailova et al., 2010) or (lipopolysaccharide) LPS (Liu et al., 2010) rather than probiotic-pathogen interactions. In one study investigating probiotic-pathogen interaction, 6 LABs were shown to inhibit *E. coli, Salmonella enterica, S. aureus, Pseudomonas brenneri, C. difficile*, and *Bacillus subtilis* (Guan et al., 2020).

T84 is another cell line derived from cancer cell lines and grown as monolayer posing same disadvantages as aforementioned cell lines. However, T84 has been shown to have high TEER properties which makes them good model to study effects of microbes on epithelial barrier function. In addition, T84 monolayers have been shown to be superior to Caco-2 as a model system of colonocytes (Devriese et al., 2017). EIEC, EPEC and enterohemorrhagic *E. coli* (EHEC) studies have used T84 cells for investigating the protective effect of probiotic strains against invasion or epithelial injury caused by pathogenic *E. coli* (Sherman et al., 2005; Khodaii et al., 2017).

Another cell line derived from epithelial cells from the rat small intestine, IEC-18, was used to show the protective effect of bifidobacteria against pathogenic invasion of entero-pathogenic *E. coli* by improving the intestinal barrier function (Yang et al., 2017).

IPEC-J2 cells are unique since they are derived from small intestinal cells (porcine origin), and most similar to humans as compared to other animal cell lines. IPEC-J2 are not transformed and mimic the normal intestinal physiology and function, and is a multi-cellular cell line with mucus producing cells that can be used for studying interactions with enteric bacteria or effects of probiotics (Vergauwen, 2015). IPEC-J2 cells have for, e.g., been used in an adhesion study, where adhesion properties of 11 *Lactobacillus sensu lato* strains were studied and *L. reuteri* and *L. plantarum* were shown to display the highest adhesion capacity to IPEC-J2 (Larsen et al., 2009).

Instead of cells in 2D models, mucus and extra cellular matrix proteins can be used as a substratum. Immobilized mucus may provide a better representation of the intestinal mucosa than some intestinal epithelial cells; under normal conditions intestinal microbes do not interact with the epithelium, but with the overlying mucus layer. Immobilized extra cellular matrix proteins may be used as a model for damaged tissue. The models have been successfully used to study the interaction between commercial probiotics and enteric pathogens (Collado et al., 2007). Since the normal intestinal epithelium consists of several different cell types like enterocytes, goblet cells, stem cells, enteroendocrine cells, and M cells a that are not accurately represented in 2D cell models, thus the three dimensional and organ-on-a-chip systems were developed that will be discussed later (Dutton et al., 2019).

### Three-Dimensional Cell Models of the Human Gut

To mimic the dynamic interactions between different players in the gut more realistically, different three-dimensional (3D) models have been developed where the cells are surrounded by extracellular matrix (ECM) that contains soluble factors, nutrients and oxygen with apical basal polarity similar to *in vivo* organization of cells. Thus, 3D systems can be used to recreate more accurate disease models (Ringuette Goulet et al., 2017; Bourland et al., 2018).

The term organoid (“organ-like”) has been used to describe a variety of 3D models that resemble *in vivo* tissues. The term intestinal organoid has been used in broad and unspecific manner, while organoids can be referred to as “enteroid” when the cells come from the small intestine and “colonoid” when cells are derived from colon (Stelzner et al., 2012). Organoids represent an attractive, physiologically relevant tool that are derived from intestinal stem cells differentiate into intestinal epithelium, mesenchyme, and lumen-like structures to form spherical structures forming spheres (Spence et al., 2011). With 3D culture system, it is possible to partially recapitulate the complexity of mammalian organogenesis *in vitro*, for example by using pluripotent stem cells (PSCs), derived from embryonic stem cells (ESCs) or induced pluripotent stem cells (iPSCs) (Werner et al., 2016).

The Rotating wall vessels (RWV) have enabled prolonged 3D culture of both cell lines and primary cells and bacterial populations and have been used for studying host- pathogen interactions. This device produces a laminar flow to enable the growth of intestinal organoids in suspension culture in conjunction with bacteria to simulate an enteric infection in a fluidic setting. However, the specific 3D surface topography of the intestine has been poorly recreated which is important for studying host-pathogen interactions (Barrila et al., 2010).

Several recent reviews have detailed the method used to generate different 3D models and the challenges involved with those techniques (Costa and Ahluwalia, 2019; Bédard et al., 2020; Zhang et al., 2020). Organoid cultures have 3D structures with villus like domains that retains cellular polarization toward tissue, and because their cells can express intestinal stem cell markers, they differentiate into all epithelial cell lineages. In short, organoids can be ever-expanding, and retain their original organ identity (Sato and Clevers, 2013). The development in organoid models has been moving quite fast and the use for organoids ranges from investigations in regenerative medicine (Wiegerinck et al., 2014) to host-microbe interaction studies (Lukovac et al., 2014) and disease modeling (Baumann, 2017). The major criticism of classical 2D cell culture systems was their inability to create the *in vivo* -like conditions ideally, which led to development of 3D systems. The 3D systems were developed with the intention of replacing animal models whenever possible, so efforts are being made to recreate as realistic models as possible (Bédard et al., 2020). However, these organoids are not appropriate to study epithelial layer maintenance that is not spherical, and it is not possible to include various stem and differentiated cell types that are present in the native intestinal tissue. The monolayer structure can be used in conjunction with other cells such as immune cells or bacteria and may also be used to study responses to different apical stimuli (Ettayebi et al., 2016; Braverman and Yilmaz, 2018). The 3D architecture is lost in this system which is the downside of using these monolayers.

Despite being advanced models, the organoids have limitations that include the ethical aspect of using live human derivatives, lack of consistency and quality control in the individual sample collected, and it is not easy to determine which factor elicited the response because of complex environment. They form closed lumen with apical side of lumen inside that is inaccessible from outside thus, they cannot be used to study the interaction of microbes with the cells (Hill et al., 2017; Bein et al., 2018) unless microinjection technique is used. Williamson et al. (2018) used a high-throughput microinjection device to inject fecal derived microbial community efficiently and reproducibly into the lumen of gut organoid. They also showed that complex microbiota communities could be transferred into the lumen and cultured for up to 4 days without changes in the microbial composition. Using the microinjection technique, Freire et al. (2019) added butyrate, lactate and polysaccharide A into the duodenal biopsies from celiac disease patients which lead to improved barrier function. While this technique opens opportunity for investigation of complex microbial communities within 3D models, microinjection technique is not easy to perform and may cause damage to the organoid structure (Heo et al., 2018). Another option is to use mechanical shearing to promote the solubilization of the semi-solid spherical structure and which can generate a polarized epithelial layer in Transwell chambers followed by microbe addition (Dutta et al., 2017; Hill et al., 2017). Using Transwell chambers also allows studying the barrier function by TEER measurement. It should be noted though that dissociating 3D models prior to infection, would disconnect their form and function causing them to lose certain phenotypes, although they may re-associate into 2D on a polarized monolayer, with similar cell types, and even a semi-3D structure that resembles intestinal folds (Braverman and Yilmaz, 2018; Kar et al., 2021). Another way to overcome the challenges to access the apical side within spheroid organoids or enteroids (Co et al., 2019) is to use a method to reverse the epithelial polarity of the enteroids so that the apical surface faces outward. Hence, no need for microinjection since microbes can be directly added to the culture media to interact with the apical enteroid surface.

Compared to 2D models there are limited number of studies involving bacteria interactions with host, particularly probiotic strains in 3D cell models. Most of the studies involving microbes have so far focused studying pathogenesis (Forbester et al., 2015; Leslie et al., 2015; Karve et al., 2017). An immensely useful technique was recently published in which the researchers have reversed the organoid polarity so that the apical surface faces the media (Co et al., 2019). This model can be used to study barrier integrity, nutrient uptake and allows us to study the microbiome-host epithelium interaction in response to diet or metabolite addition (Rubert et al., 2020). However, this approach can only be used for aerobic bacteria or for cell free supernatant from strict anaerobes.

Enteroids have been used to study host response to pathogens such as Enterohemorrhagic *E. coli* (In et al., 2016), enterotoxin producing *E. coli* (Rajan et al., 2020) and even cholera-toxin (Zomer-van Ommen et al., 2016). Aoki-Yoshida et al. (2016) used murine intestinal enteroids to study the effect of *L. rhamnosus* GG. Some commensal bacteria used in conjunction with enteroids include *Akkermansia muciniphila* and *Faecalibacterium prausnitzii* (Lukovac et al., 2014), however they used the supernatants of these commensal bacteria. Han et al. (2019) used human organoids to demonstrate the protective effect of *L. rhamnosus* GG to epithelial barrier dysfunction. They used fecal supernatants from intestinal bowel syndrome patients to induce barrier damage. *L. reuteri* D8 was shown to repair the epithelial damage caused by TNF-α treatment in the co-culture of mouse intestinal organoids and human lamina propria lymphocytes, leading to improved intestinal barrier function and epithelial layer proliferation (Hou et al., 2018). Interaction of non-pathogenic strain of *E. coli* with cells in organoids derived from human intestinal stem cells showed stable host-microbe symbiosis that lead to improved epithelial barrier (Hill et al., 2017). The anti-cancer activity of *L. fermentum* was compared in 3D vs. 2D cell model and it was concluded that the 3D models are more appropriate for studying anti-cancer benefits of probiotic strains (Lee et al., 2019). While this is not a probiotic-pathogen interaction study it directly compares the effects of a probiotic strain in 2D vs. 3D.

3D cultures that arrange themselves during proliferation into sphere-like formation, are called spheroids. Caco-2 spheroids were found to be a good model for getting broad information on the possible interaction mechanisms between host and bacteria of importance for food safety when used for evaluation of the adhesion/invasion ability of *Lactobacillus sakei 1* and *Listeria monocytogenes* (Pereira et al., 2021). Gastric dendritic cells control the adaptive response to *Helicobacter pylori* infection and when spheroid cultures of primary gastric epithelial cells have been infected with *H pylori* to study the response of the gastric epithelium (Sebrell et al., 2019), thus it could also be used for studying probiotics.

Recently another *in vitro* 3D model for culturing human gut microbiota and for assessing the production of stable and long-lasting biofilms was developed. The model consists of a biofabricated electrospun structure of gelatin where human fecal microbiota can be cultured on the scaffolds and the microbial biofilm can be monitored and quantified over time (Biagini et al., 2020).

A further model is HuMIX (human-microbial crosstalk); a sophisticated multi-channel model using co-culture of Caco-2 cells with *L. rhamnosus* GG or *Bacteroides caccae* (microbes cultured under anaerobic conditions), with which it was shown that the two microbial species generated different metabolic and immune responses (Shah et al., 2016). On the downside, the model has two separate chambers for intestinal and microbial cells with a thin membrane separating the two channels and there is no pulsatile flow unlike, e.g., in the microfluidic “Gut-on-a-Chip” (Kim and Ingber, 2013).

### Microphysiological/Chip-Based Models of the Gastrointestinal Tract

One of the most recent advances in *in vitro* intestinal cell models is organs-on-a-chip. These microfluidic based models mimic complex multi-organ and multi-layered systems *in vivo* and enables the exploration of pathophysiological features of human microbial infections (Baddal and Marrazzo, 2021). The structural and functional integrity can be maintained for many days allowing for experimental designs that explore change in biological response over a period (Barrila et al., 2018). Several human gut-on-chips models have been developed to better mimic the complexity of the *in vivo* intestinal epithelium (Kim et al., 2012, 2016a,b; Kasendra et al., 2018). However, only some gut-on-chip models, e.g., by the Wyss Institute have incorporated microbes for studying specific interactions of bacteria with host cells. This microfluidic model stimulates the formation of villus-like structures by Caco-2 cells and can be combined to simulate exposure to microbes and human cells in a simulated sub mucosa (Bein et al., 2018). The model maintains aerobicity for the tissue culture cells while the microbes are in an anaerobic environment; thus, better mimicking the intestinal situation better than traditional 2D tissue culture models. This way changes in microbial response due to certain stimuli can be studied; which bacteria are metabolically dominant or what is the transcriptional profile of microbes in response to cell stimuli (Jalili-Firoozinezhad et al., 2019; Poceviciute and Ismagilov, 2019).

The advantages of organ-on-chips include a 3D environment that mimic tissue structure and incorporates cells lines or stem cells. They exhibit *in vivo* like properties and cells are able to differentiate into specialized cell types. The chips are microfluidic systems that allow simulation of cellular microenvironment (Ingber, 2016). However, they are laborious and require substantial expertise before they can be used. They are technically challenging and utilize small volumes and cell numbers. The methods developed so far to perform downstream analysis from organ-on-a-chip sample are limited. Real-time monitoring for these chip-based models is not easy and mostly end time analysis is used. Although, one organ-on-a-chip model in combination with organoid technologies where primary patient-derived colonic epithelial cells was used to recapitulate mucus bilayer formation does allow real time monitoring (Sontheimer-Phelps et al., 2020). The use of chip-based models by industry has encouraged companies to improve the reproducibility and manufacture process of chips. The manufacturing and experimental cost of using the chip-based models is still quite high. The components needed for experiment are so far of disposable material which increases the cost even further. Additional challenges include sample collection from chip, which can cause change in concentrations of metabolites when samples are withdrawn during experimental run. The samples size that can be obtained from these models is small because of small numbers of cells which may be a constraint for downstream analysis. Like all *in vitro* models the benefits of organs-on-a-chip need to be weighed against disadvantages when designing the experiment. As more laboratories implement these models for bacterial interaction studies the protocols will start to become standardized between labs thus leading to more physiologically relevant answers.

### *In vitro* Gastrointestinal Models

Several different models mimicking the human gastrointestinal tract and especially the colon have been developed during the last few decades. There are batch, semi-continuous and continuous models and different conditions can be applied such as fasted or fed state, as well as different disease. In contrast to the above mentioned static and dynamic biofilm models, gastrointestinal models tend to focus on planktonic microbes rather than immobilized microbes.

Batch cultures are the simplest form of an *in vitro* digestive model since they are usually composed of a single vessel containing the appropriate media that is inoculated with the probiotic and pathogen, and then incubated under specific temperature and atmosphere mimicking the gastrointestinal conditions, before analyzes are made. In addition, fecal derived bacteria can also be mixed to the batch cultures (Tejero-Sariñena et al., 2013; Piatek et al., 2020). Although this is a simple model, it is still an informative tool for larger screening.

The more advanced models have been built for different purposes. While some models mimic the whole gastrointestinal tract like, SHIME (Van de Wiele et al., 2015) others represent a specific part like colon by EnteroMix (Lamichhane et al., 2016), or combination of two different models like TIM-1 and TIM-2 (Minekus, 2015). Shortly, SHIME consists of five reactors and allows culturing of the intestinal microbiota over a longer period of time and it is possible to assess probiotic properties of food or ingredients after a 2–3-week administration of the probiotic product. EnteroMix consist of four glass vessels representing the different parts of the colon. While SHIME has a feeding rate of 140 mL 3x/day, EnteroMix has a feeding rate of 24 ml per day. TIM-1 represents the stomach to small intestine, while TIM-2 simulates the colon.

The different models apply variable setups and different types of active components and concentrations. Moreover, different fluid flows are applied, e.g., batch vs. semi-continuous vs. continuous flow. However, one thing many of these models have in common for having a representative gut microbiota, is the use of fecal samples. Currently this is the best solution, although, the fecal samples do not provide the information about in probiotic -pathogen interaction in the small intestine, where most of the digestive process are carried out.

Many of the models are built for assessing how different food components affect the gut microbiota and hence enable monitoring or quantification of microbial changes as well as microbial metabolites in different parts of the gastrointestinal tract. The models can also be used for studying antibiotic induced dysbiosis and the restoration of the gut microbiota (Liu et al., 2020).

One example of a pathogen that has been investigated with or without probiotics in more complex digestive *in vitro* models is *C. difficile*. Although *C. difficile* can be present in the adult commensal gut microbiota, antibiotic treatment increases the risk of infection due to *C. difficile* (Zhang et al., 2015). This organism has been studied in e.g., the EnteroMix and PolyFermS models. In the EnteroMix model the pathogen was included as vegetative cells (Forssten et al., 2015), while the PolyFermS used both vegetative cells and spores (Fehlbaum et al., 2019).

As discussed above, the effect on the gut epithelium is important and hence, the digestive models can be combined with different cell models, preferably with all the layers of the mucosa. None of the models make it possible to directly study the diseased states of the gut or interactions between the aerobic and anaerobic gut microbiota and the host intestinal epithelium that is important when studying health effects. In addition, it should be possible to culture each part without losing their characteristics, i.e., the model should include adequate oxygenation and nutrients to the cell medium, as well as physiological shear and have a biochemical environment that enables the crosstalk between epithelium, immune system and the gut microbiota (Costa and Ahluwalia, 2019; Jalili-Firoozinezhad et al., 2019).

### *In silico* Models

Since probiotics have different mechanisms of action in the gut, one possibility to investigate their interactions with gut microbiota and pathogens is by *in silico* approaches and this is an emerging field (Geng et al., 2021). Mathematical models have been used for, e.g., to predict the conditions under which probiotics may be successful in promoting the health of infants suffering from NEC (Arciero et al., 2010) while genome scale metabolic models (GEMs) can be used for evaluating the metabolic potential of a probiotic as well as the interaction with other organisms in the gut microbiota (Choi et al., 2020).

### *Ex vivo* Models of Functional Tissues

*Ex vivo* models are made up of functional live tissues including complex cellular environments cultured outside the host. Human intestinal explant technology allows maintenance of whole organ or a part of it in culture by using specialized conditions (Randall et al., 2011; Tsilingiri et al., 2013). These tissue explants have the obvious disadvantages of difficulty in acquiring tissue samples and the short explant viability; regardless these models have been used to study interaction of microbiota with host. Thus, these methods are unsuitable for high throughput screening. Few studies have used this technology to determine the interaction of intestinal microbiota and the effect of different microbes (Randall et al., 2011; Bergström et al., 2016). Overall, *ex vivo* systems contain added complexity and functional crosstalk between many different cell types that are not generally found in *in vitro* systems (Roeselers et al., 2013). A limitation of current *ex vivo* systems is that they cannot be used in anaerobic environment which is a requirement for studying the intestinal niche. The field for *ex vivo* model is developing and given the interest, available organ types and advances in real time monitoring techniques it is likely improvements for *ex vivo* models will be seen.

The InTESTine model uses fresh healthy porcine intestinal tissue from gastrointestinal tract, mounted horizontally to an oxygenated incubator. The model was designed for drug discovery research and can be used with or without the microbiota incorporated and hence it can be used to study bacteria-host interaction. The model includes a mucus layer allowing better culturing of mixed bacteria community. Another *ex vivo* model, the Ussing chamber has been developed to utilize live mammalian tissues or cell on snap-well dishes. A drawback with Ussing chambers is that the tissue remains viable for a limited time and cannot be used for studies longer than approximately 5 h (Thomson et al., 2019). Ussing chambers have been used, e.g., for studying infection of Caco-2 monolayers by EIEC and the impact of a probiotic mixture (*B. longum, L. acidophilus*, and *E. faecalis*), or single strains, and the probiotic treatment was shown to enhance resistance to the EIEC invasion as well as reduced the secretion of proinflammatory cytokines (Shi et al., 2014).

Other examples of *ex vivo* tissue culture models include the calf ileal epithelium model (Frost et al., 1997) the human intestinal *in vitro* organ culture (IVOC) model (Haque et al., 2004). IVOC can also be used with Ussing chamber (Van Krimpen et al., 2014), the *ex vivo* intestinal mucosa model (Tsilingiri et al., 2013), and the *ex vivo* immature human intestinal tissue model (Newburg et al., 2016). These models mimic the organs during infection. However, the *ex vivo* models have their own challenges such as their short lifespan, laborious set up, inconsistency in experiments, and limited availability of cells.

### “Simpler” Animal Models for Microbiota Research

Lower vertebrates and invertebrates can also be used for studying probiotic-pathogen interactions (Newton et al., 2013; Douglas, 2019). These systems have microbiomes with lower taxonomic diversity than in mammals. For example, the nematode, *Caenorhabditis elegans* although a well-established model for some areas, it is still in its early stages as a model to study microbiome interactions. The use of invertebrates like the honey bee (*Apis mellifera*) (Zheng et al., 2018), *Ciona robusta* (Liberti et al., 2021), fruit flies (*Drosophila melanogaster*), and greater wax moth (*Galleria mellonella*) are also an emerging field.

There are several reasons why *C. elegans* can be an excellent model to replace vertebrates in research conducted on viable models. There are physiological and functional similarities between microvilli in human intestines and *C. elegans* and 40% of the genes of *C. elegans* are homologous to humans (Lai et al., 2000). *C. elegans* has a short life ranging from 2 to 3 weeks which make it possible to study the effect of host-microbe on the whole lifespan. The worms are transparent which makes visualization of organs or colonization of microbes a possibility using microscopy. The *C. elegans* can be fed fluorescently tagged bacteria and the interaction and colonization of bacteria can be imaged in real time in the viable worm body (Rezzoagli et al., 2019). Some studies have investigated the effect of probiotics such as lactobacilli, *Bacillus* and bifidobacteria, on metabolism, signaling, pathogen-specific defense responses, and lifespan of *C. elegans* (Zanni et al., 2015; Kumar et al., 2020; Poupet et al., 2020). Also immune modulation by selected probiotic strains has successfully been investigated in *C. elegans* models (e.g., Kim and Mylonakis, 2012; Zhou et al., 2014; Lee et al., 2015).

Despite all the advantages of research using *C. elegans*, there are some important aspects that need to be considered; like, e.g., the impact of its own microbial community and the interaction of its species with its host to determine which changes should be expected when researcher manipulate its microbiota by feeding bacteria of interest. Another consideration before using the *C. elegans* models for microbiome studies for human research is that although *C. elegans* overlap with human profile at the phylum level, it is completely different at the genus level. While, Firmicutes and Bacteroidetes comprises a major proportion of human microbiota, Proteobacteria are the main colonizers in *C. elegans* (Dirksen et al., 2016; Samuel et al., 2016).

Another non-vertebrate *in vivo* model that can be used for host microbiota interaction studies is *D. melanogaster*. The fly model can be used to perform studies validating the effect of probiotics on living organisms. This model has advantages like being inexpensive and breeds rapidly as well as high throughput screening capabilities including tools for studying host-microbe interaction as it has been used as a model in pathogen research (Trinder et al., 2017; Poupet et al., 2020). A limitation of using *D. melanogaster* is that their intestine is physiologically quite different from mammals but the gastrointestinal physiology, anatomy and signaling are highly conserved (Apidianakis and Rahme, 2011). *D. melanogaster* has a lower microbial diversity than humans, with only 1–30 species and *Lactobacillus sensu lato* and Acetobacter are the most dominant ones (Wong et al., 2011; Chaston et al., 2014). In addition, unlike *C. elegans* the microbial strains cannot be fed to the *D. melanogaster* but must be injected which eliminates the initial stages of the infection process. *D. melanogaster* has been used to some extent for probiotic-pathogen studies. When live microorganisms (*Bacillus cereus, Candida inconspicua, Issatchenkia hanoiensis, and Klebsiella* sp., mixed in an artificial diet) were fed to *D. melanogaster* for 1 day prior to infection with *Aspergillus flavus* the mortality of the flies was significantly decreased as compared to controls infected with *A. flavus* alone (Ramírez-Camejo et al., 2017). Others have shown that *L. plantarum* decreased the survival rate of *Diaporthe* FY infected Drosophila (Su et al., 2019), as well as mitigated survival deficits after *Serratia marcescens* septic infection (Daisley et al., 2017).

A lesser used invertebrate model is the wax moth *Galleria mellonella* (Cutuli et al., 2019). This model has the limitation that there are fewer investigative techniques available for it. However, a big advantage is that it can survive at both 25 and 37°C which makes it a promising tool (Nathan, 2014). It has been used as a model to study bacterial pathogenesis (Ramarao et al., 2012; Mukherjee et al., 2013). In a study by Vilela et al. (2015) larvae were used as a model for pathogenic yeast infection with *C. albicans* and co-infection with a strain of *L. acidophilus*. An advantage is that unlike the previously mentioned non-vertebrate models the immune mechanisms of *G. mellonella* is quite similar to humans (Nathan, 2014). The genome of *G. mellonella* has not been completely sequenced yet and there is need to standardize the methods between different laboratories (Mukherjee et al., 2013; Nathan, 2014). Another study showed the *in vitro* long-term colonization of two type strains of *L. plantarum* both *in vitro* on colon cells lines (Caco-2 and HT-29) and *in vivo* in *G. mellonella*. This study also showed the reliability of *G. mellonella* oral administration model as a first-line screening tool for *in vitro* to *in vivo* translation (Venditti et al., 2021). Another study successfully demonstrated *G. mellonella* as an *in vivo* model to assess the protection conferred by probiotic microorganisms against gastrointestinal pathogens. The antibacterial activity of probiotic strains, *L. rhamnosus* GG and *Clostridium butyricum* Miyairi, was tested against three enteric pathogens causing infection in *G. mellonella*: *Salmonella enterica* Typhimurium, EPEC or *Listeria monocytogenes* (Scalfaro et al., 2017).

## Future Perspective

While there are regulatory aspects for novel probiotic species (EFSA, 2020), as well as regulation (2017/746) on *in vitro* diagnostic medical devices (IVDR), i.e., any medical device which is a reagent, reagent product, calibrator, control material, kit, instrument, apparatus, piece of equipment, software or system, whether used alone or in combination, intended by the manufacturer to be used *in vitro* for the examination of specimens, including blood and tissue donations, derived from the human body, solely or principally for the purpose of providing information on, e.g., concerning a physiological or pathological process or state or to determine the safety and compatibility with potential recipients (Dagher et al., 2019). However, no specific regulation for non-medical *in vitro* methods exists.

Due to the differences between models and the difficulty to compare the results between the different models and different teams, an effort made to improve this lack was the COST INFOGEST network (Brodkorb et al., 2019; Colombo et al., 2021). The network aimed to develop a static model that is easy to set up and that could be applied for the large research community, i.e., “to harmonize *in vitro* static systems that simulate digestive processes by defining key parameters and conditions.” Thus, by combining this static model with other dynamic models, more *in vivo* like conditions can be achieved, since it would be important to develop intestinal *in vitro* models that can be specifically used for specific research questions and to improve the translation of *in vitro* to *in vivo* research within the gastroenterology area.

## Conclusion

In conclusion, the main challenge with the different gastrointestinal models is to have physiologically relevant models that mimic the *in vivo* response. In addition, it is highly appreciated if results can be achieved precisely in as short time as possible, and that it is possible to perform analysis of supplements or foods with different composition. The ideal model includes all essential features of the biological counterpart it is intended to represent, and as all above described methods have their own advantages and limitations (Table 1), thus each model needs to be assessed before use to answer a specific research question.

## Author Contributions

MA: writing—original draft preparation. AO and SF: writing— review and editing. AL: review and editing. All authors have read and agreed to the published version of the manuscript.

## Conflict of Interest

The authors were all employees of Danisco Sweeteners Oy, IFF Health & Biosciences (Kantvik, Finland). IFF produces and markets probiotics.

## Publisher’s Note

All claims expressed in this article are solely those of the authors and do not necessarily represent those of their affiliated organizations, or those of the publisher, the editors and the reviewers. Any product that may be evaluated in this article, or claim that may be made by its manufacturer, is not guaranteed or endorsed by the publisher.
